# Predictive topology refinements in distributed stream processing system

**DOI:** 10.1371/journal.pone.0240424

**Published:** 2020-11-05

**Authors:** Muhammad Hanif, Choonhwa Lee, Sumi Helal

**Affiliations:** 1 Division of Computer Science and Engineering, Hanyang University, Seoul, Republic of Korea; 2 School of Computing and Communications, Lancaster University, Lancaster, United Kingdom; King Abdulaziz University, SAUDI ARABIA

## Abstract

Cloud computing has evolved the big data technologies to a consolidated paradigm with SPaaS (Streaming processing-as-a-service). With a number of enterprises offering cloud-based solutions to end-users and other small enterprises, there has been a boom in the volume of data, creating interest of both industry and academia in big data analytics, streaming applications, and social networking applications. With the companies shifting to cloud-based solutions as a service paradigm, the competition grows in the market. Good quality of service (QoS) is a must for the enterprises, as they strive to survive in a competitive environment. However, achieving reasonable QoS goals to meet SLA agreement cost-effectively is challenging due to variation in workload over time. This problem can be solved if the system has the ability to predict the workload for the near future. In this paper, we present a novel topology-refining scheme based on a workload prediction mechanism. Predictions are made through a model based on a combination of SVR, autoregressive, and moving average model with a feedback mechanism. Our streaming system is designed to increase the overall performance by making the topology refining robust to the incoming workload on the fly, while still being able to achieve QoS goals of SLA constraints. Apache Flink distributed processing engine is used as a testbed in the paper. The result shows that the prediction scheme works well for both workloads, i.e., synthetic as well as real traces of data.

## Introduction

With the evolution of cloud computing from a set of promising virtualization and data center technologies to a centralized paradigm for the delivery of the computing as a service to customers (like other utilities such as water, gas, and electricity) in a pay-as-you-go manner, adaptation of the technology by enterprises is growing fast by days, and so is the number of cloud-based companies offering cloud services to end customers. This has subsequently resulted in an exponential rise of distributed streaming frameworks, capable of dealing with “big data”, “fast data”, and data streams alike, allowing for quick and characterized decisions. The applications of these engines can be seen in several types of data, including but not limited to, social media posts, search queries, sensor logs, etc, an example of which could be on a local news broadcast, where social media feeds regarding a specific event or tragedy are analyzed in seconds [1], and Google’s Zeitgeist pipeline can detect any exceptional queries within search parameters on the event in question quickly [2].

To fulfill these analytical applications needs, several distributed stream processing systems have been developed, which can process large and fast continuous streams of data on the fly and respond to the user queries within near real-time. Examples of prominent streaming frameworks include: Millwheel developed by Google [2], Apache Storm [3], Spark Streaming [4], Ptail and Puma created by Facebook [5], Microsoft’s Naiad [6], and Apache Flink [7]. These systems, despite their differences in terms of design and technical detail, do have a few similarities, namely in terms of: a) Data Parallelism: distributed stream processing systems exploit parallelism to scale the processing to a cluster level. Data parallelism essentially splits a larger dataset into more manageable subsets, through either physical or logical partitioning, which then allows the tasks to be executed in parallel across the subsets. b) Incremental Processing: most of the distributed stream processing systems have the competence to process data incrementally, as opposed to batch processing where each operator processes all the data, then forwarding the gathered data onto the next operator, in a repeated loop, resulting in a significant delay of the final result.

Apache Flink is considered to be one of the most auspicious, open-sourced, distributed real-time stream frameworks to date, having the capacity to deal with rapidly large data streams in a flexible and dependable way. Flink has meritoriously done for stream processing what Hadoop [8] has done for batch processing. Flink is built on the principle of working coherently over unbounded data streams to be executed as a stream of fault-tolerant data flows and related streaming applications such as fraud detection in real-time banking transactions, real-time stream analytics of business applications, iterative algorithms like graph processing and machine learning. Recent distributed streaming frameworks have already corrected several of the issues plaguing big data applications, but there are still lingering issues [9], namely with the topological readjustment of the operators used in these systems, usually resulting in cluster performance and QoS degradation.

Achieving QoS marks is crucial for meeting Service Level Agreements (SLAs) in terms of latency, throughput, or application performance with the customers. It is a primary reason of heavy investment taking place in the field for the enterprises that provide streaming as a service to the end customers. More customers would likely stick with the enterprises that provide satisfactory QoS. However, the process is further complicated due to uncertainty and unpredictable situations at run-time. Recent research efforts revealed a deficiency in dealing with dynamism inherent in distributed stream processing systems, which includes:

- Topology reconfiguration: rectifying a topology when dealing with an active application, can usually lead to the obstruction of said application’s execution, including all interconnected elements of the application. For instance, topology rebuilding might be necessary to fulfill a user request of incrementing or decrementing certain parameters.
- Error Estimation: system administrators and users can often underestimate or overestimate their application needs because of a lack of understanding of requirements due to complexities. As a result, it becomes extremely hard for users to find a right combination of parameters that can suitably fit current and anticipated application workload.
- Dynamic Workload: streaming applications receive data from a large number of sources (like sensors, system logs, IoT devices, etc.). Thus, highly variable load spikes in data can occur, depending on the day and time of the year as well as application popularity. Thus estimating the workload behavior (event and data arrival pattern, I/O behavior, distribution of service time, and network usage) is crucial for the performance and optimum utilization of the whole system.
- Workload assessment knowledge: there is a habitual performance degradation and poor QoS, as topologies are mapped to nodes regardless of the knowledge of the workload, as by default in the streaming processing systems. This leads to over- or underutilization of resources of individual nodes or even the whole system in some cases.

To guarantee a service level agreement in terms of application performance, latency, and error rates, what is required of stream processing systems is the ability to connect system configuration and application performance. Based on the prediction of incoming workload fluctuations, the topological refinement of the system should be able to be adjusted accordingly. Several state-of-the-art distributed stream processing frameworks support the ability to manually change the operator distribution and topology. However, to the best of our knowledge, none of the major frameworks has implemented the mechanism to automatically refine the topology with respect to the changes in the incoming workload. There are two cases we should consider to make topology adjustments. First case is when the incoming data rate exceeds the system capacity to process it and unprocessed data accumulate, causing a back pressure and in due course making the system inoperable. Second case is when the incoming data rate is very low and the buffer needs to wait for the incoming data to be available and then fired to process it accordingly. As a result, the refined topology would allow the system to yield higher performance.

In a previous work [10], we introduced an architecture for topology refining using a simple prediction mechanism. One key component of the proposed architecture is our topology-refining scheme for Flink framework. However, one missing piece of the puzzle in the work was a suitable mechanism for workload prediction. In this paper, we present our TRS(Topology Refining Scheme) system capable of refining and re-adjusting the topology of streaming processing systems on the fly at run-time based on autoregressive and moving average workload prediction models. The key contributions of this work are as follows. First, we proposed a stream pipeline system which takes workload prediction and user SLA into account in order to select a physical topological plan to run streaming applications. We designed a workload prediction module using a combination of Moving Average (MA) model [11] and Autoregressive (AR) model [12] with added feedback step so as to predict the incoming workload of the system. The prediction module can be considered as a special case of ARIMA model [13]. Furthermore, a hybrid model of support vector regression (SVR) and ARIMA model is employed, which yields better prediction results than other single models due to the fact that it is capbable of capturing both linear and nonlinear features. Most importantly, the prediction is then used to refine the topology of the system. Finally, we conducted an evaluation study of the system using both real and synthetic workloads.

The rest of the paper is as follows. Section 2 explicates distributed stream processing system model, Section 3 introduces the architectural design of an adaptive stream processing system. Section 4 provides an evaluation of our system and Section 5 gives a brief taste of related work in the area. Finally, Section 6 represents a conclusion of our work, as well as any future steps that we might take for this project.

## Distributed stream processing system

With the advancement and adaptation of cloud computing and related technologies by enterprises, we have seen a subsequent rise in the number of relevant big data applications in a variety of fields such as online banking systems, real-time streaming analytics, online traffic analysis systems, Internet of things (IoT), social media feeds, etc. These systems are creating real-time unbounded data on a large scale. To handle such a vast amount of seemingly limitless data in an efficient and expansive manner, a host of streaming processing systems emerged, including Dataflow model [14], Samza [15], Storm, and Flink. These frameworks deal with any and all arriving, real-time streams that is distributed to each of the nodes in the cluster. Modern state-of-the-art distributed stream processing allow the job graph’s operators to be duplicated throughout the cluster, decreasing latency and raising the throughput.

**Fig 1:**
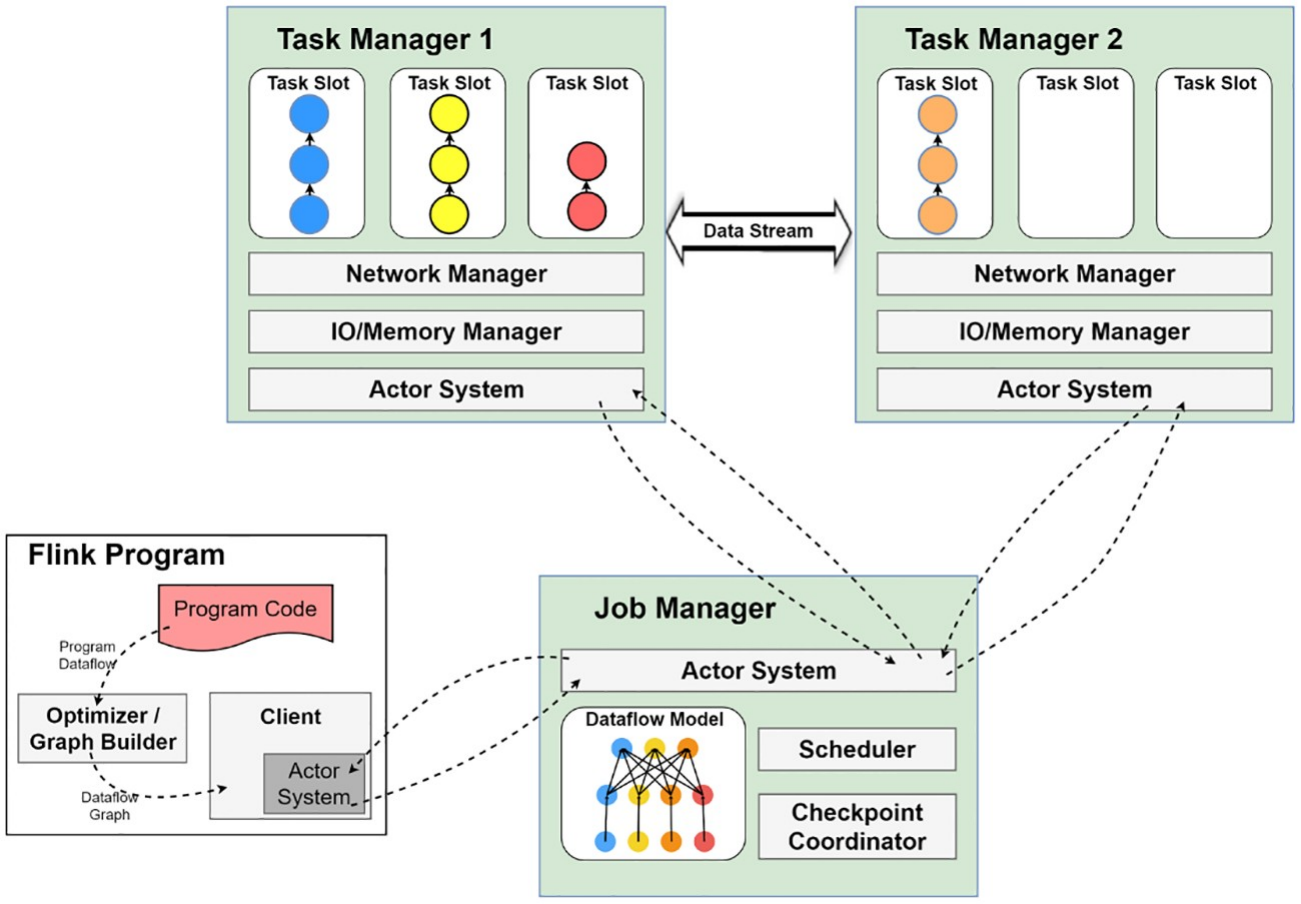
Program code transformation and operator distribution by job manager in Apache Flink framework.

Typically a distributed streaming system is comprised of nodes working together as a cluster to run applications over it in a distributed manner. The resources for the executions of tasks in a Flink cluster are Task slots. Task managers and individual worker nodes all have at least one or more task slots, with each slot having the capacity to execute a pipeline of parallel tasks [16]. Every pipeline is composed of a number of sequential tasks, such as map, reduce, join, filer, sink, and union functions. As illustrated in Fig 1, Flink has the ability to run both batch and streaming application’s tasks simultaneously. The batch task is treated as a special case of streaming tasks. The user code coordinates with the job manager, with the actor system acting as a medium. The job manager then delegates each of the available operators in the task managers located in the cluster to execute any operations in their respective task slots.

Let’s assume that we have a Flink application that must run on the cluster. The application’s code is analyzed and outputed as a dataflow graph, utilizing either the default or the user-specified parallelism for all operators and functions. The graph is then pipelined through to the client section of the system, where the client forwards the refined program outline, as a dataflow graph, towards the job manager. The communication between client and job manager happens through their actor system. The job manager then translates the received plan into a physical execution plan, allocating operators based on the set parallelism. As seen in Fig 1, the pipeline contains the order of Source-Map-Reduce-Sink operators. With this particular case, we assigned the Map Function a parallelism of three, as well as a Reduce Function parallelism of four. The job manager then gives task pipelines to task managers through an actor system. Each task manager then distributes the tasks into the available task slots to be executed accordingly.

**Fig 2:**
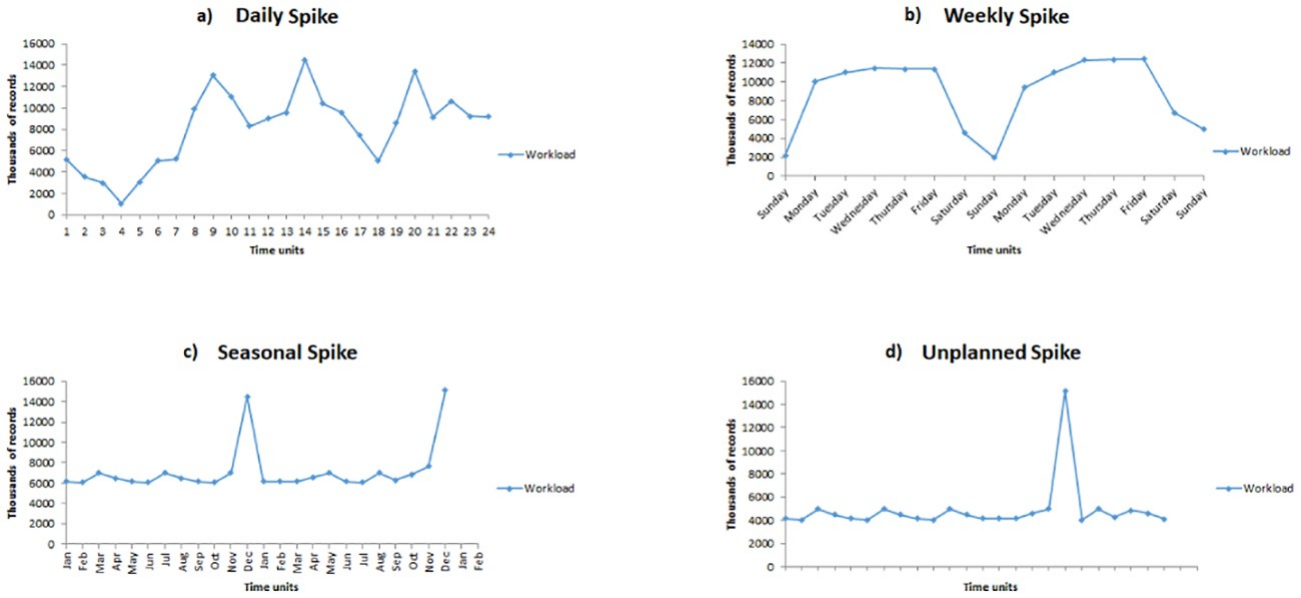
Workload Spikes: a) Daily spikes are in the mornings or in the evening. b) The weekly are in the weekdays starting from Monday evening till Saturday morning. c) Seasonal spikes could be around Christmas and New Year holiday’s season. d) Unplanned spikes can occur at any time of the year.

The majority of streaming engines have the capability to utilize event-time windows, mirroring the actual occurrence of said events, but in many realistic situations, such as the New York City Yellow Taxi Trip Records [17] and the German Credit Cards dataset [18], there always exists the chance of a spike in the workload cycle, which can vary as a daily, weekly, seasonal or even unexpected cycle occurs, as seen in Fig 2. We generated synthetic workload to mimic different patterns of spikes and show how the system adapts itself accordingly in section 4. Daily spikes in the cycle typically arise in the mornings or evenings, typically the busiest times of day, while the weekly spikes usually happen on business days (Monday evening through to Saturday morning). Seasonal spikes typically occur over the holidays, like Christmas, while unexpected spikes can happen at any point in time across the year. To handle this immense workload, a system is required to have the capability of scaling upwards or downwards in terms of the operator’s parallelism in the pipeline, depending on any arriving data streams.

Multiple modern, distributed streaming engines have the capability to manually alter the operator’s distribution topology. This capability notwithstanding, according to our research, no framework has managed to provide us with the required capabilities or features needed to automatically alter the operator’s distribution topology, factoring in the arriving workload. Recognizing this deficiency, we developed a streaming processing system architecture capable of predicting the incoming workload and refining the system topology according to the near future prediction of the incoming workload.

## Adaptive topological refining system

### Architectural design

One of the key challenges of stream processing systems is elasticity, which enables the underlying system to be dynamic and adaptive towards the fluctuation of incoming event and data streams. However, adapting the system to the incoming workload requires an insight into the system operation and incoming workload. Also, with the Stream Processing as a Service (SPaaS) that enables users to build and operate custom managed streaming applications, the service providers may also be liable for not delivering the minimum required QoS. The main idea behind SPaaS is to allow the user to focus on business application logic, while the platform provides the scale, operations, and domain expertise.

**Fig 3:**
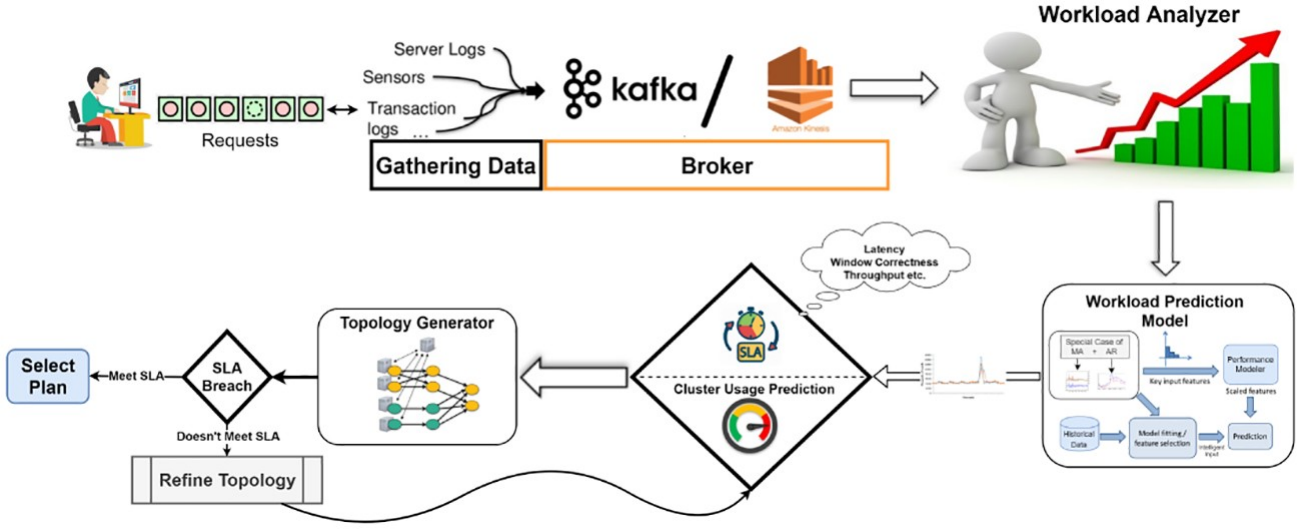
System architecture: System administrator and users push requests to the system through event and data gathering component of the systems, which then tunnel it to the brokering agent. The broker passes it to the workload analyzer that in turn passes the analysis results to the workload prediction model. The prediction results are then checked with the cluster usage and SLA constraints and are then passed to the topology generator to generate topology. If the resulting topology meets SLA requirements while considering cluster usage prediction, then the execution plan is selected; otherwise topology is refined, and the process is repeated.

One approach that has been explored for other cloud services such as PaaS, SaaS, etc. for years is based on workload prediction. Accurate predictions of a user’s future service requests enable the service provider to meet the QoS targets according to the SLA agreement. In this paper, we focus on seasonal request pattern applications such as requests to a Web or online gaming servers, and e-commerce [19–21]. To overcome the unpredictability in workload patterns and minimize estimation errors in forecasting incoming streams of data, we proposed an adaptive topology refining mechanism. As diagrammed in Fig 3, our system architecture is composed of user/system administrator request queue, data steam input gathering module, brokering module, workload analyzer module, workload prediction modeling module, topology generator, and physical topology selection module. The input gathering module gathers all incoming input from various sources (IoT sensors, transaction logs, etc.), per the users request. The collected data is then sent as a data stream to a data broker, like Kafka or Amazon Kinesis, which then siphons the input data to the system’s workload analyzer module.

The module utilizes the recently arrived data to approximate the metric amounts to be used in the system’s analytical algorithms, before forwarding it to the prediction module. Then, the workload prediction module, which is based on the implementation of a hybrid model of SVM and ARIMA time series process (detailed in the next section), produces predictions accordingly. The developed predictions are then verified according to the cluster usage and SLA constraints, before being sent to the topology generator, which will develop a topology according to a concrete and refined understanding about the arriving workload. The system verifies it in accordance with the SLA agreement, and if all the conditions are met, an execution plan is chosen to suit the topology. If not, the job manager petitions for an enhancement of the topology. The resource manager provides a number of resources to aid in the execution of the application, utilizing the open slots in the task managers. This procedure is redone, until the coveted results are obtained, and QoS objectives are met to match the SLA agreement set by the user.

### Problem definition

**Fig 4:**
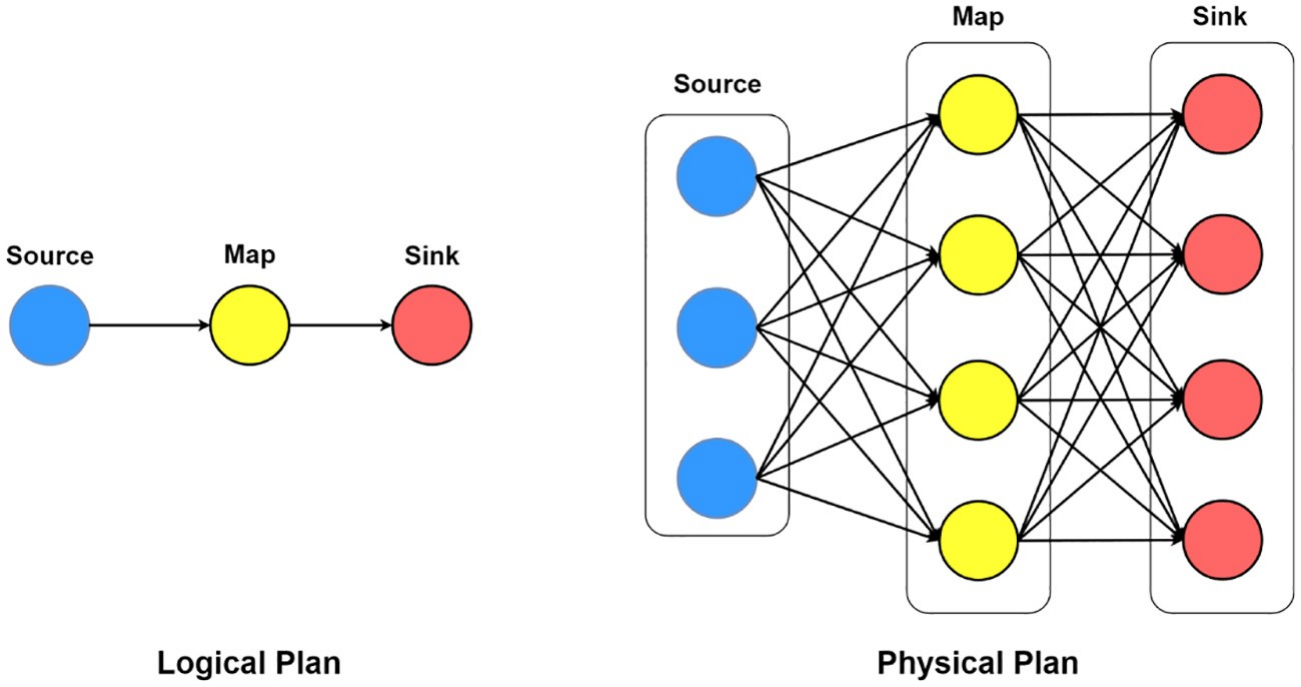
Logical and physical execution plan represented through directed acyclic data flow graphs.

Most of the state-of-the-art distributed stream processing systems execute data-parallel applications over a shared-nothing cluster. The logical representation of such application is in the form of directed acyclic graph *G* = (*V*, *E*), where *V* represents vertices as operators and *E* represents edges as data dependencies between these operators. Vertices with no upstream operators are source operators and those with no downstream operators attached are sink operators. Vertices with no upstream operators are source operators and those with no downsream operators attached are sink operators. DSPS systems translate logical DAG to a physical execution plan that maps operators to provisioned resources. A logical topology is the logical execution plan of the topology which is then translated into a physical topology that specifies the physical instances or worker threads of each logical operation. Let graph *G*′ = (*V*′, *E*′) represents the physical execution plan. *V*′ are the operator instances of the corresponding vertex in *V* and edges are the incoming data links. Fig 4 illustrates the logical execution plan and its corresponding physical execution plan through directed acyclic data flow graphs with a source, map, and a sink operator, this phenomenon is known as chaining. In case of distributed execution, Flink system chains operator subtasks togather into tasks. Each thread execute a single task accordingly. Chaining operators into tasks reduces the overhead of thread-to-thread handover and buffering, and help achieve better trade-off between throughput and latency. Source executes with three instances, map and sink with four instances each.

Suppose we have a logical DAG data flow with *s*_1_, *s*_2_, …, *s*_*n*_ source operators, and *e*_1_, *e*_2_, …, *e*_*m*_ edges, with each source operator with the rates corresponds to it as *ν*_1_, *ν*_2_, …, *ν*_*k*_. The default and maximum parallelism of the operators are defined through configuration files or set in the code program using the defined API of the Apache Flink core. Source operators of the distributed data applications such as server logs, sensors, stock market feeds, or transaction logs generate records at a rate *ν*_*k*_, defined by application. For application to have optimized throughput, all the operators must have the ability to process the data upstream operators without any delays or bottlenecks. Our topological refining scheme targets workload changes based on the prediction results and tries to refine the topology with minimal increase in latency or data loss.

### Operator mapping technique

Most of the distributed stream processing systems currently circumvent optimal operator mapping with the physical machine through only supporting pre-defined operator locations with pinned operators in the network. This leads to situations where the system administrator is responsible for efficient operator placement. This situation is infeasible for a dynamic and large scale distributed system with thousands of queries to process. Some of the distributed systems such as Medusa [22] try to solve this by balancing the incoming workload among the nodes. This approach is only good for a single data centre and leads to poor performance in the presence of a wide-area network. PIER [23] build a distributed database on top of DHT with the location of operators and corresponding relational tables, leading to effectively mapping operators with resources randomly through hashing.

The main challenge with the operator mapping mechanism is that systems usually do not have any knowledge or heuristic measures about the incoming workload. Therefore, random placement of the operators with the default parallelism does not guarantee the system to maintain SLA agreement. We design our operator-mapping algorithm to satisfy conditions such as scalability, adaptive and can maintain SLA agreement to be more efficient. It is essential for it to be scalable in case of both resources as well as operator distribution capabilities. It has the ability to adapt to changes in conditions and workload behavior. In addition, it does not breach SLA agreement while mapping different operators to resources. The system generates logical topology based on the workload prediction model and check whether it will breach SLA agreement if deployed. If not, then it will generate physical topology based on this logical one and will recheck the SLA breach, if it meets the plan will be deployed. Otherwise, the topology refinement module will be called to refine the topology according to the changes in both the cluster usage and available resources.

### Workload prediction

The challenge of workload prediction is mainly handled through two different approaches: reactive approach and proactive approach. Reactive approach is when the system reacts to the changes based on predefined thresholds. Proactive approach is to take preemptive measures to imminent changes before its occurrence through future load prediction. Proactive is achieved through methods that can monitor and forecast workload capture the relationship between application QoS targets and workload pattern changes to refine and readjust the topology of the system at run-time. In order to achieve more accurate predictions in time series forecasting, a combination of key approaches has been adapted. A time series may have seasonal patterns as well as non-linear patterns. Seasonal patterns can be modeled by traditional statistical methods like Autoregressive Integrated Moving Average (ARIMA) model, while non-linear patterns can be modeled using nonlinear models such as support vector regression (SVR). The workload prediction module adapts its prediction using a hybrid model of SVR and a variation of autoregressive integrated moving average (ARIMA) time series process [13]. We will first briefly explain time series modeling and then will cover the fitting of the model to our situation.

#### Linear modeling

A time series is defined as a sequence of consecutive data points indexed or graphed with respect to time. Our assumption about time is that it is a discrete variable called *X*_*t*_ representing the observation or data node at time t, and ∈_*t*_ represents the zero-mean random noise term at time t. Moving average model, MA(q), refers to the moving average model of order q and considers the process in Eq 1:

$$\begin{array}{c} X_{t}=\sum_{i=0}^{q}\beta_{i}\in_{t-i}+\in_{t} \end{array}$$ (1)

where *β*_*i*_ is a coefficient. Similarly, the autoregressive model, i.e. AR(k), refers to the autoregressive model of order k and is represented under the conditions to satisfy Eq 2:

$$\begin{array}{c} X_{t}=\sum_{i=0}^{k}\alpha_{i}X_{t-i}+\in_{t} \end{array}$$ (2)

The above equation can be detailed in a way that it assumes each *X*_*t*_ is a noisy linear combination of previous k data points. The only difference between this and the traditional multiple regression model is that *X*_*t*_ is regressed based on past values of *X*_*t*_. After a combination of MA(q) and AR(k) model, autoregressive moving average model, i.e., ARMA(k, q), arises, which provides a flexible modeling platform. The notation ARMA(k, q) refers to the model with k autoregressive terms and q moving-average terms. The *X*_*t*_ is represented through Eq 3:

$$\begin{array}{c} X_{t}=\sum_{i=0}^{q}\beta_{i}\in_{t-i}+\sum_{i=0}^{k}\alpha_{i}X_{t-i}+\in_{t} \end{array}$$ (3)

where ∈_*t*_ are zero-mean noise term. In order to make the process stationary, constraints need to be applied to the weight of AR(k) part. An invertible and stationary ARMA(k,q) model can be represented either as an infinite autoregressive model, i.e., AR(∞) or an infinite moving average model, i.e., MA(∞). It is known that the ARMA(k,q) with comparison to AR(∞) and MA(∞) has the feasibility to generate stationary stochastic processes with an only finite number of parameters [24].

As evidence suggests, modern time series real-world data is not realizations of a stationary process. In such cases, to manage such strong correlations effectively is through a differential mechanism. For example, computing first-order differences of *X*_*t*_ using Eq 4:

$$\begin{array}{c} \nabla X_{t}=X_{t}-X_{t-1} \end{array}$$ (4)

Moreover, second-order differences in *X*_*t*_ using Eq 5:

$$\begin{array}{c} \nabla^{2}X_{t}=\nabla X_{t}-\nabla X_{t-1} \end{array}$$ (5)

In case the ∇^2^*X*_*t*_ sequence satisfies an ARMA(k,q) model, then *X*_*t*_ satisfy Autoregressive integrated moving average, i.e., ARIMA(k,d,q) model, which can be calculated using Eq 6:

$$\begin{array}{c} \nabla^{d}X_{t}=\sum_{i=0}^{q}\beta_{i}\in_{t-i}+\sum_{i=1}^{k}\alpha_{i}\nabla^{d}X_{t-i}+\in_{t} \end{array}$$ (6)

Eq 6 is parameterized by three terms *k*, *d*, *q*, and weights vector *α* belongs to $R^{k}$ and *β* belongs to $R^{q}$. The ARMA(k,q) becomes a special case of ARIMA(k,d,q) with the differences of order zero. Predictions with ARIMA(k,d,q) can be viewed as reversion of different order of differential process. For example, if a time series sequence *X*_*t*_ satisfies ARIMA(k,d,q), then the d-th order differential at time *t* + 1 can be predicted as ∇^*d*^*X*_*t* + 1_ and prediction of data point at time *t* + 1 will be calculated as $\tilde{X_{t}}$ in Eq 7:

$$\begin{array}{c} \tilde{X_{t}}=\nabla^{d}\tilde{X_{t}}-\sum_{i=1}^{d-1}\nabla^{i}X_{t-i} \end{array}$$ (7)

The prediction module receives historical workload in a preparatory step so that the ARIMA(k,d,q) to be fit on them. After the system becomes operational, it predicts from one to ten-time interval in advance. In our case, we select a single time interval for simplicity purposes. The length of the time interval can be adjusted as application-specific to fit best accordingly. The prediction results are being kept in a buffer which updates itself by adding new reading and removing the oldest reading in the buffer. The values of p and q are determined through analyzation of autocorrelation occurrences of historical data, respectively. The randomness of the data stream is determined through the autocorrelation plot. The autocorrelation values approach zero for time-lagged values in case of randomness. Otherwise, some autocorrelation values approach 1 or -1. The autocorrelation plot is a combination of time lags on the horizontal axis and autocorrelation coefficient *R*_*h*_ on the vertical axis calculated as in Eq 8:

$$\begin{array}{c} R_{h}=\frac{C_{h}}{C_{0}} \end{array}$$ (8)

where *C*_*h*_ is the auto-covariance function defined as in Eq 9:

$$\begin{array}{c} C_{h}=\frac{1}{N}\sum_{t=1}^{N-h}\left(X_{t}-\bar{X}\right)\left(X_{t-h}-\bar{X}\right) \end{array}$$ (9)

where *N* is the number of samples, and $\bar{X}$ is an average of samples *X*_*t*_, t = 1,2,3….N, and *C*_0_ is the variance function and is defined as in Eq 10:

$$\begin{array}{c} C_{0}=\frac{1}{N}\sum_{t=1}^{N}{\left(X_{t}-\bar{X}\right)}^{2} \end{array}$$ (10)

#### Nonlinear modeling

The SVR model is based on the structured risk minimization (SRM) principle that performs minimization of the upper bound of the generalization error [25]. Suppose ${\left\{X_{i},Y_{i}\right\}}_{i=1}^{l}$ be a training set where $X\in {ℜ}^{d}$ is the *i*-th input vector, $Y_{i}\in ℜ$ is the *i*-th prediction output of *x*_*i*_, *d* is the embedding dimension of the time series, and *l* is the number of training samples. SVR tries to find the best function from a set of possible functions in the form as in Eq 11:

$$\begin{array}{c} \{f|f\left(X\right)=W^{T}X+b,w\in {ℜ}^{d},bℜ\} \end{array}$$ (11)

Where *w* is the weight vector estimated by the minimizing the regularized risk function as in Eq 12 and *b* is bias or threshold.

$$\begin{array}{c} \frac{1}{2}{\left||w|\right|}^{2}+C\sum_{i=1}^{l}L(Y_{i},f\left(X_{i}\right) \end{array}$$ (12)

It is significant to minimize the regularized risk to find the best function, where *C* > 0 is a regularized factor, ||.|| is a 2-norm, and *L*(., .) is a loss function. In order for the SVR to perform a nonlinear mappings into a higher dimensional space, it needs to use kernels as in Eq 13.

$$\begin{array}{c} f\left(X\right)=\sum_{i=1}^{l}\left(\alpha_{i},{\alpha_{i}}^{*}\right)k\left(X_{i},X\right)+b \end{array}$$ (13)

Where *α* and α* are lag-range multipliers and *k*(*X*_*i*_, *X*) is a kernel function.

#### Hybrid modeling

In order to capture both the linear and nonlinear features of the workload, a hybrid model is a good alternative for prediction of most of the real-world workload scenarios. ARIMA and SVMs models have abilities to model features in linear or nonlinear domains. Therefore, a hybrid model of ARIMA component and SVM component called ARIMA-SVR is proposed to improve the overall forecasting performance. The hybrid ARIMA-SVR model can be represented as in Eq 14.

$$\begin{array}{c} Z_{t}=Y_{t}+N_{t} \end{array}$$ (14)

Where *Y*_*t*_ is linear component and *N*_*t*_ is the nonlinear component of the model. Both *Y*_*t*_ and *N*_*t*_ are estimated from the dataset. $\tilde{N_{t}}$ is the predicted value of the ARIMA model at time *t*. Suppose *ε*_*t*_ is the residual at time *t* as obtained from the ARIMA model. Then, it can be represented as in Eq 15.

$$\begin{array}{c} \varepsilon_{t}=Z_{t}-\tilde{Y_{t}} \end{array}$$ (15)

The residuals are modeled by the SVMs and can be represented as in Eq 16.

$$\begin{array}{c} \varepsilon_{t}=f\left(\varepsilon_{t-1},\varepsilon_{t-2},\ldots .,\varepsilon_{t-n}\right)+\Delta t \end{array}$$ (16)

where *f* is a nonlinear function modelted by SVM and Δ*t* is the random error. Therefore, the hybrid forecast is as in Eq 17.

$$\begin{array}{c} \tilde{Z_{t}}=\tilde{Y_{t}}+\tilde{N_{t}} \end{array}$$ (17)

where $\tilde{N_{t}}$ is the predicted value from Eq 16.

The historical workload information is fit to the model which leads to a desired prediction. The prediction is in turn given to the topology generator to have an intelligent decision accordingly. Furthermore, reducing the effort of the system administrators requires better prediction results of the workload behavior where prediction module gradually learns from historical data pipeline using machine learning techniques. Such direction of using advance machine learning techniques to predict the workload behavior may be a good path for future research in this regard.

### Performance metrics

#### a) Throughput

One of the crucial tasks of the distributed stream processing system is to find optimal operator placement or select physical nodes that should host the operators and map them with each other. An operator placement metric quantifies the quality of a given placement or mapping. There are challenges with factoring mapping operators to physical resources in a distributed cluster including re-usage of existing operators, application query performance, and workload knowledge.

Distributed streaming framework provides users with a rich metric API set. The average throughput per second is calculated by getting the number of output records at the sink operator by a remote procedure call to the API function as in Eq 18 below:

$$\begin{array}{c} Throughput=\frac{Ň}{T} \end{array}$$ (18)

where *Ň* is the result as the number of output records at the sink operator return by the API function, while T is the time since submission of the application to the platform.

#### b) Latency

Latency is one of the complex metrics to be estimated in streaming applications, considered a difficult metric to gather especially on the scope of a big data stream. Therefore, it is achieved through sampling of records from the mainstream periodically and estimating each sample’s latency individually as needed. Sampling of all records is conducted, as the inclusivity of all elements in the calculation will affect the system’s performance. Individual records are marked at source operators using a watermarking mechanism and the sink operator that uses this extra information in the watermark of the records so that only marked records will be used for the calculation.

In this manner, the sink operator can identify the exact records for the latency computations. Record marking at a source operator can be done in recurring intervals, or through a random selection algorithm. The Job Manager (master node) calculate the latency through the following Eq 19:

$$\begin{array}{c} Latency=t_{finish}-t_{start} \end{array}$$ (19)

where *t*_*finish*_ is the finish time of the marked sample and *t*_*start*_ is the arrival time of the sample record in the execution pipeline.

The proposed system gets the current latency and compares it with the target latency based on the service level agreement. In case the current latency is greater than that of target latency, the system alerts the actor system in the resource manager about the SLA breach. It then signals the resource manager to refine topology by increasing the number of running threads. In case the current latency is less or equal to the target latency, the system gives a green signal to the actor system about the current topology generated by the topology generator and an execution plan is selected based on the current topology. The system repeats the procedure for the throughput as well; it compares the current throughput with the target throughput based on user SLAs. In case the current throughput is less than the target throughput, the system refines topology. In the end, the resource manager selects a physical execution plan based on the generated logical topology for the system.

## Implementation and evaluation

**Fig 5:**
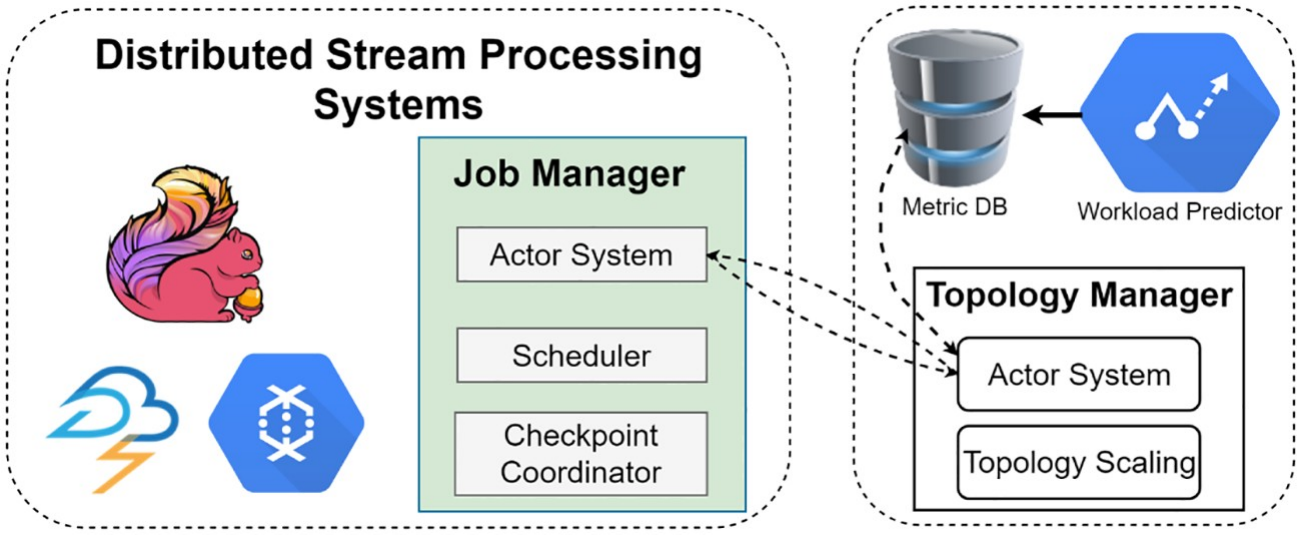
Program code transformation and operator distribution by job manager in Apache Flink framework.

Topological Refining Scheme is a standalone plugin type of process with the ability to be integrated as a black box within other state of the art systems. Those systems must have the ability to collect and send information about certain things such as records produced, read, and waiting time and so on. We choose Apache Flink as our test bed as it has the ability to collect metrics like produced records, read records, processed records, frequency of input and output etc. as well as easy to extend its runtime with very low overhead. In addition, it has the capability to adjust its parallelism or number of threads that run in parallel on the fly. The high-level integration architecture is shown in Fig 5. The workload predictor estimates the metrics and stores it in a database. The Topology Manager monitors this metric repository and updates the parallelism if new updates in the metrics occur. It checks the metric repository periodically every two minutes. We selects a two minute period for the sake of simplicity as well as to avoid extra overhead from the system. The topology manager implemented an actor system to communicate with the job manager’s actor system. Every time an update occurs in the topology, the Job manager halts the system and takes a snapshot of the job state, and redeploys the job with its refined topology.

**Fig 6:**
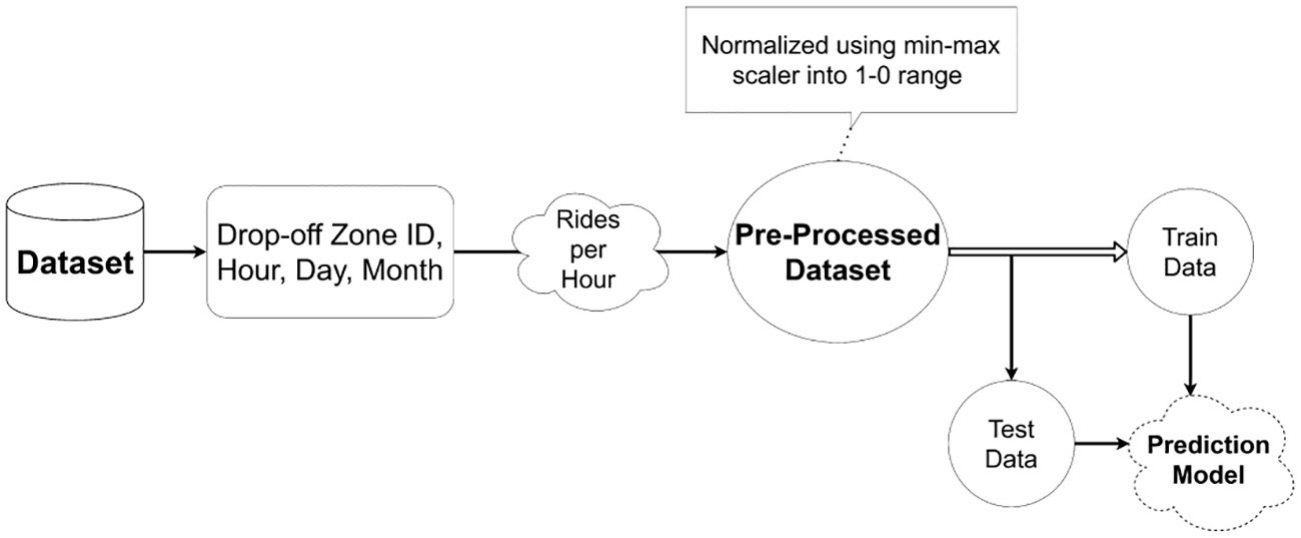
Data flowchart for prediction of hourly, daily, and weekly drop-offs for certain zones or the entire city in TLC dataset.

In this article we used two different benchmark datasets which represents real world application scenarios called New York City Yellow Taxi Trip Records [17] and the German Credit Cards dataset [18]. The NYC TLC trip record data is provided by the New York City Taxi and Limousine Commission. The data set is comprised of Yellow, Green, and For-Hire Vehicle (FHV) types of trip records. We performed our experimentation using the Yellow taxi trip records, because this data is representative of real-world applications and scenarios. The data has features capturing the following details: pick-up and drop-off dates/times, pick-up and drop-off locations/zones, itemized fares, trip distances, driver-reported passenger counts, payment types, and rate types. For this dataset we did experimental study to predict hourly, daily, and weekly drop-offs for certain zones or entire city based on the input query as illustrated in Fig 6. The dataset is given as an input stream to the system which then extracts certain features like hour, day of the week, month, and drop-off zone for each taxi ride. The number of rides for each time zone is then calculated, after which the data is normalized using min-max scaler to a range of 0 to1 accordingly. Afterwards, the dataset is divided into a 70 percent train dataset and a 30 percent test dataset. The second dataset used in our evaluation study is the popular Statlog German Credit (SGC) dataset. The data is available at the UCI Machine Learning Repository [18]. We used the numeric verion of the dataset. It includes 1000 borrowers records grouped in two different classes of accepted applicants of 700 instances and of rejected or bad applicants of 300 instances. All the instances have 20 input attributes including 13 categorical fields and 7 numerical fields as detailed in Table 1. We transformed the categorical attributes into numerical ones.

**Table 1:** Features applied to assess the credit risk in the German credit dataset.

Features	Description	Type	Values Range
G1	Status of existing checking account	Categorical	1-4
G2	Duration in months	Numerical	4-72
G3	Credit history	Categorical	0-4
G4	Purpose	Categorical	0-10
G5	Credit account	Numerical	276-18424
G6	Savings account	Categorical	1-5
G7	Present employment since	Categorical	1-5
G8	Instalment rate in % of disposable income	Numerical	1-4
G9	Personal status and sex	Categorical	1-5
G10	Other debtors/guarantors	Categorical	1-3
G11	Present residence since	Numerical	1-4
G12	Property	Categorical	1-4
G13	Age in years	Numerical	19-75
G14	Other instalment plans	Categorical	1-3
G15	Housing	Categorical	1-3
G16	Number of existing credits at this bank	Numerical	1-4
G17	Job	Categorical	1-4
G18	No of people being liable	Numerical	1-2
G19	Have telephone or not	Categorical	1-2
G20	Foreign worker	Categorical	1-2

**Fig 7:**
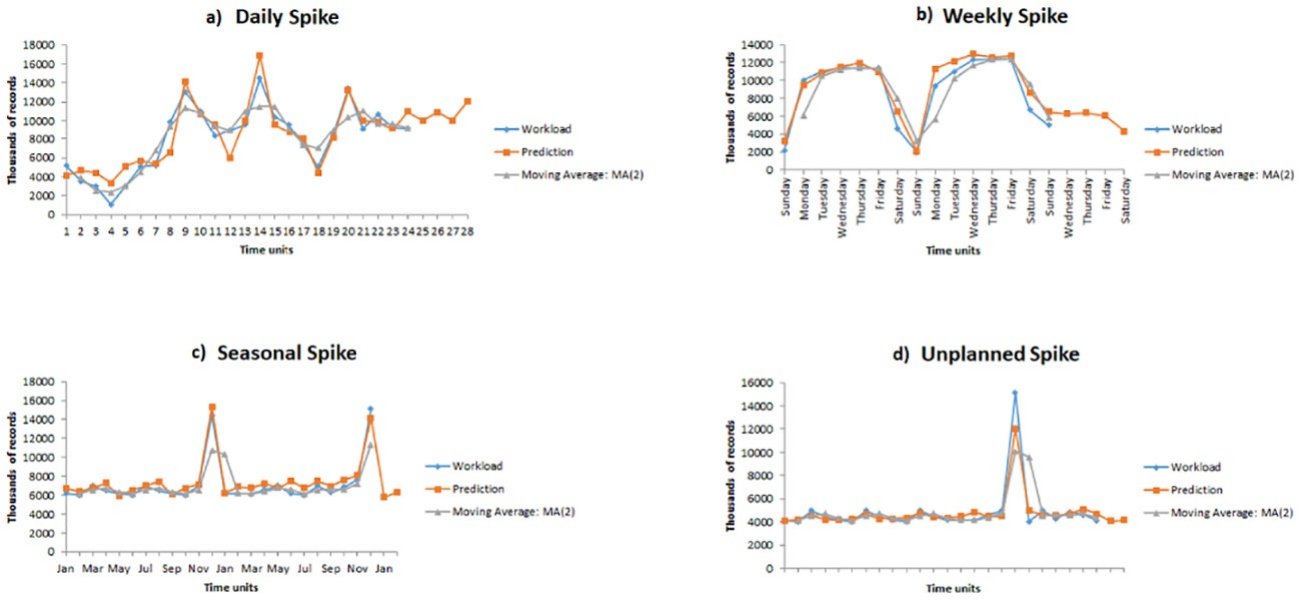
Synthetic workload spikes: The moving average results shows its occurrence till 24 hours, while the prediction is shown for three hours in the near future as well. The moving average is used to smooth the data for the prediction algorithm to fit with different spikes such as daily, weekly, seasonal, as well as unplanned ones. The prediction for spikes shows its adaption to the ups and downs in the workload.

The system evaluation occurs in two folds with the experimentation of prediction system using both synthetic and real traces of data to examine and evaluate the effectiveness and robustness of the module as well as checking its effect on the topological refining system. As for the synthetic workload evaluation, we generated synthetic workload to mimic different possibilities of spikes as mentioned in an earlier section that can occur in real-world workload including daily, weekly, seasonal, and unplanned spikes. Fig 7 shows that in each type of spikes plotted, horizontal axis plots time while the vertical axis plot the numbers of requests or records per time unit. In each different spike plot, three things are plotted, i.e., workload, its moving average to smooth out the original workload, and its prediction results. In the daily cycle (Fig 7a), the time units used are hours plotted at the horizontal axis against a number of records or events per minutes. There are spikes around mornings, noon, and evening time, and the forecasting for each spike is handled and predicted accordingly. In case of weekly spikes (Fig 7b), the time units used are days plotted at horizontal axis against number of requests at vertical axis. The figure shows that there is an increase in the number of events starting on Monday till Wednesday, after Wednesday it starts declining till the weekend and the process repeat itself for the upcoming weeks. Fig 7b shows that moving average smooth over the workload and then the system predict the cycle accordingly. In the case of seasonal spike (Fig 7c), the time unit used is months plotted against number of events occurrence and is predicted as expected. Moreover, in Fig 7d is the unplanned spike with random time units plotted against number of data records. The prediction algorithm was able to predict the unplanned spike in the synthetic workload as well.

**Fig 8:**
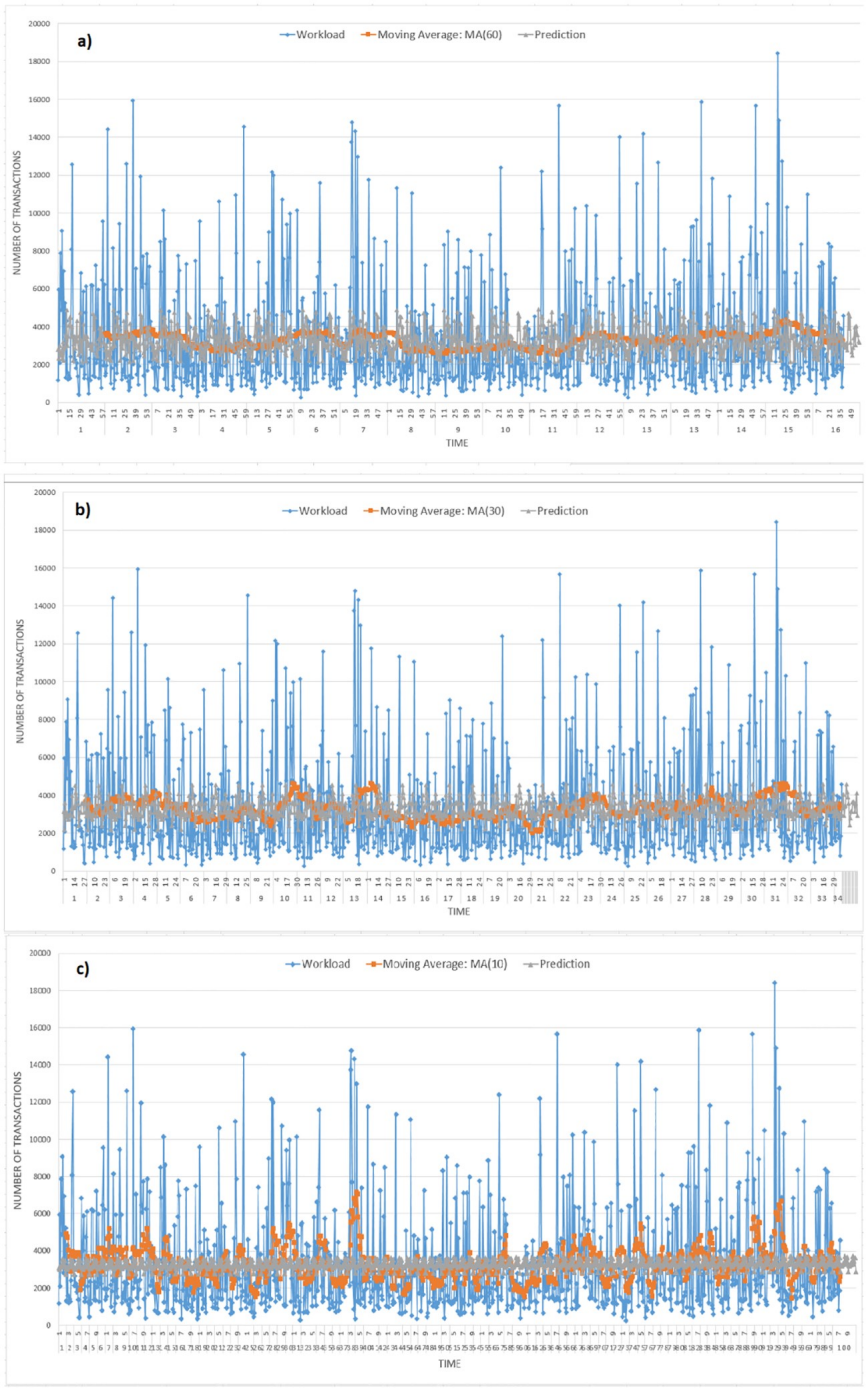
Number of requests vs. time, prediction is shown with varying values of moving averages 60, 30, and 10.

For our second set of experiments, we use German credit card dataset [18]. We tried to evaluate the data system with different experimental settings, including changing the moving average value ranging from 60 to 10. We use nine-tenth of the data for training purposes. The event occurrence rate corresponding to such periods are transformed into a time series process and the values of p, d, and q are defined. The model updates itself upon new events arrivals with the placement of new events at the front of data stream and removing the oldest events from the end of the data stream, and the fitting process repeats accordingly. The output of the prediction module is a number and a different confidence percentile range can also be extracted subsequently for each value. Fig 8 presents actual workload values, i.e., values observed in data stream with its different moving averages 60, 30, and 10 and predicted values. The moving average shows the smoothing seasonality and trend component of the graph. Various error metrics are used in the evaluation of the accuracy of the prediction.

We ran linear regression on the workload and the values are presented in Table 2. The regression analysis output shows us the fitness of the linear regression equation on the dataset. Multiple R represents the correlation coefficient and measure the strength of linear relationship. R Square is the coefficient determination and shows the number of points falling on the regression line. Adjusted R Square adjusts the model terms in numbers. Standard Error is the estimated value of standard deviation. Observation shows the sample size. In case of ANOVA, SS represents the sum of squares, MS is the value of regression SS over the degree of freedom, F is overall F test for null hypothesis, and Significance F is significance associated P-value. As per our experiments, we did not use this part of the figure in a meaningful way. The last part of the figure shows different values for the intercept and slop. Coefficients give least squares estimate, the standard error is least-square estimate of standard error, T Stat is T statistic for alternative hypothesis vs. null hypothesis, and the last is lower and upper boundaries for confidence interval. The linear regression equation is as in Eqs 20 and 21:

$$\begin{array}{c} y=mx+b \end{array}$$ (20)

$$\begin{array}{c} y=slope*x*intercept \end{array}$$ (21)

**Fig 9:**
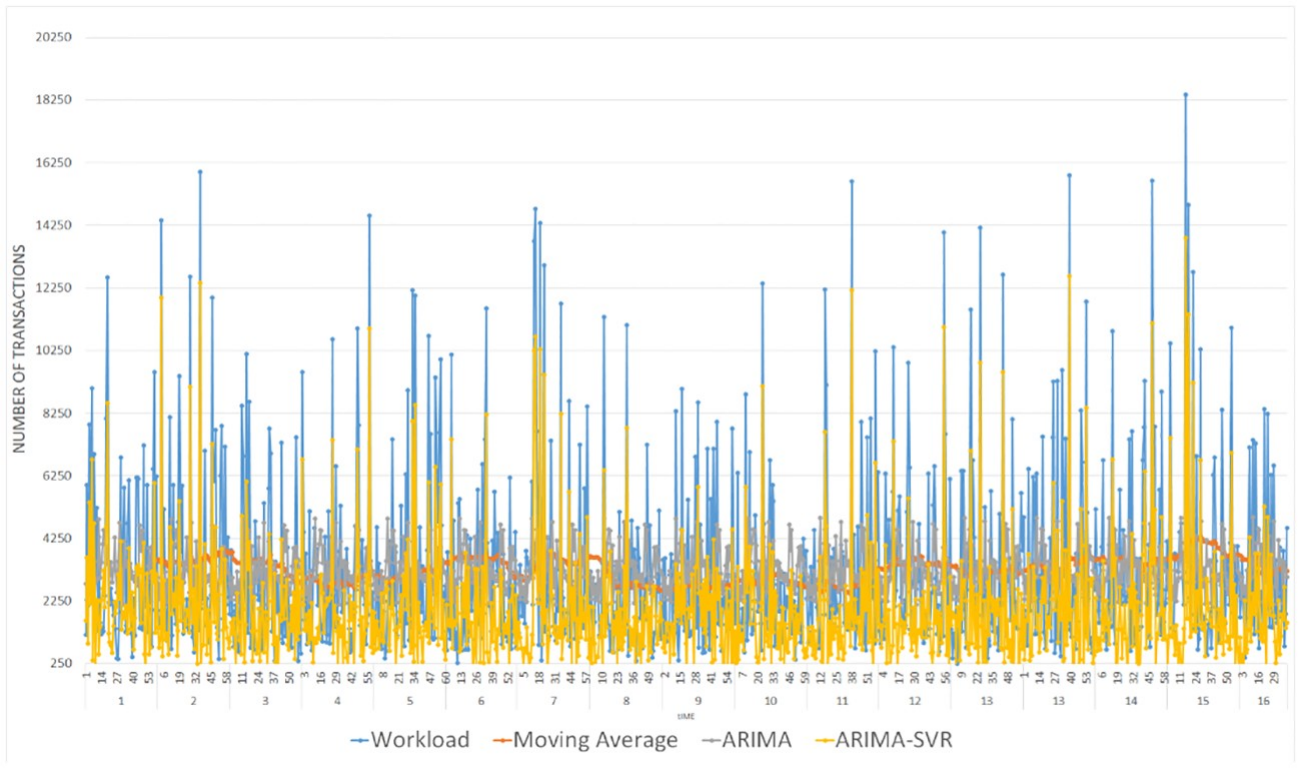
Comparison of MA, ARIMA and ARIMA-SVR model’s prediction accuracy.

Fig 9 plots point-to-point comparison of actual and predicted values. As for the moving average, it was with its variation of 60, ARIMA (2,3,2) model to forecast, and a hybrid model of ARIMA-SVR to predict the workload fluctuations accordingly. The simulation results show that ARIMA (2,3,2) model has been found to be the more parsimonious which is also considered to be sufficient for the residual analysis. The ARIMA-SVR model yields better forecasting results than other models, which can be attributed to to the hybrid model’s ability to capture both linear and nonlinear features.

**Table 2:** Linear regression results.

Regression Statistics					
Multiple R	0.0039569				
R Square	1.565E-05				
Adj. R Square	-0.0009863				
Standard Error	2708.3369				
ANOVA					
	df	SS	MS	F	Significance F
Regression	1	114623.31	114623.31	0.015627	0.900543
	Coefficients	Std. Error	t Stat	P-value	Lower 95.0%
Intercept	3279.2197	171.41881	19.129870	9.732E-70	2942.83
Slop	0.0370875	0.2966836	0.1250068	0.900543	-0.5451077

**Fig 10:**
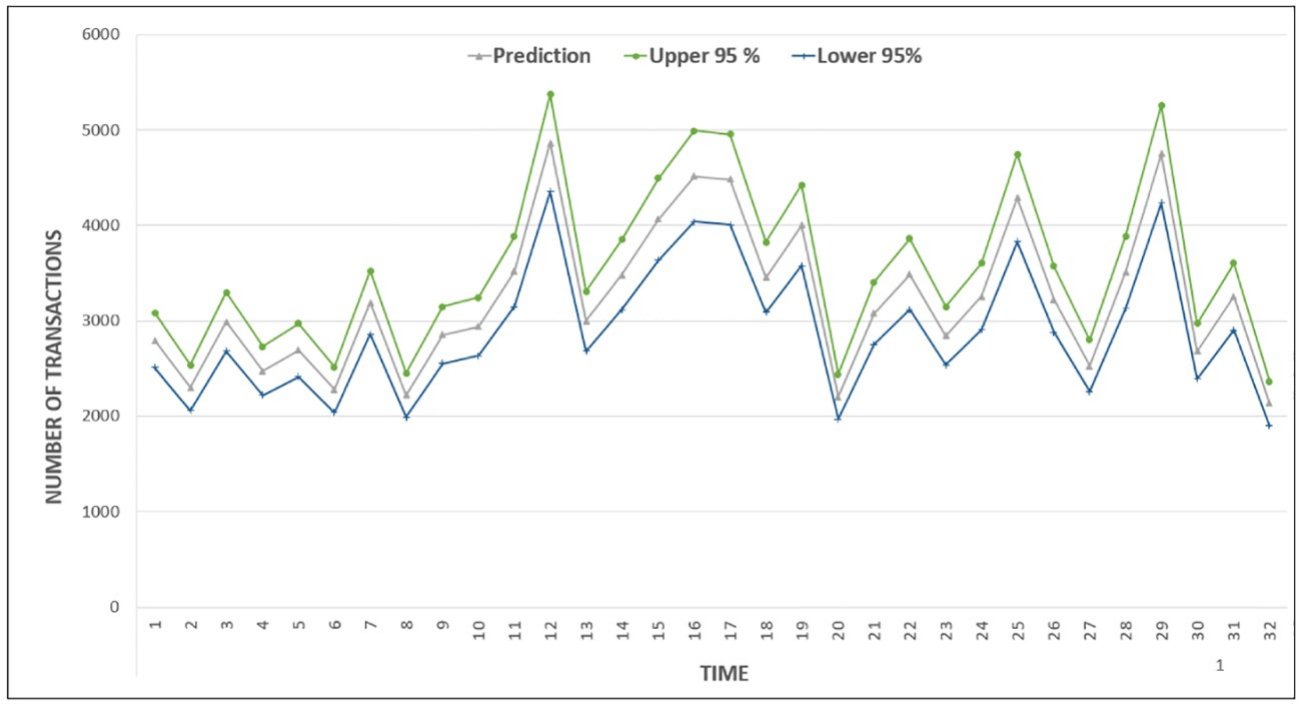
Prediction with an upper and lower limit of 95% confidence interval.

Fig 10 plots both actual prediction values and range (upper and lower boundaries) of a confidence interval for 95 percent. The low 95 percent and high 95 percent contains the limits for the 95 percent confidence interval for the prediction. The confidence intervals output can be used in cases of tradeoff decision between QoS for SLA and utilization or utilization and response time. Fig 9 also confirms that although using high limits of confidence intervals minimizes the underestimation occurrences, it causes a decrease in prediction accuracy by making it close to 78 percent on average.

**Fig 11:**
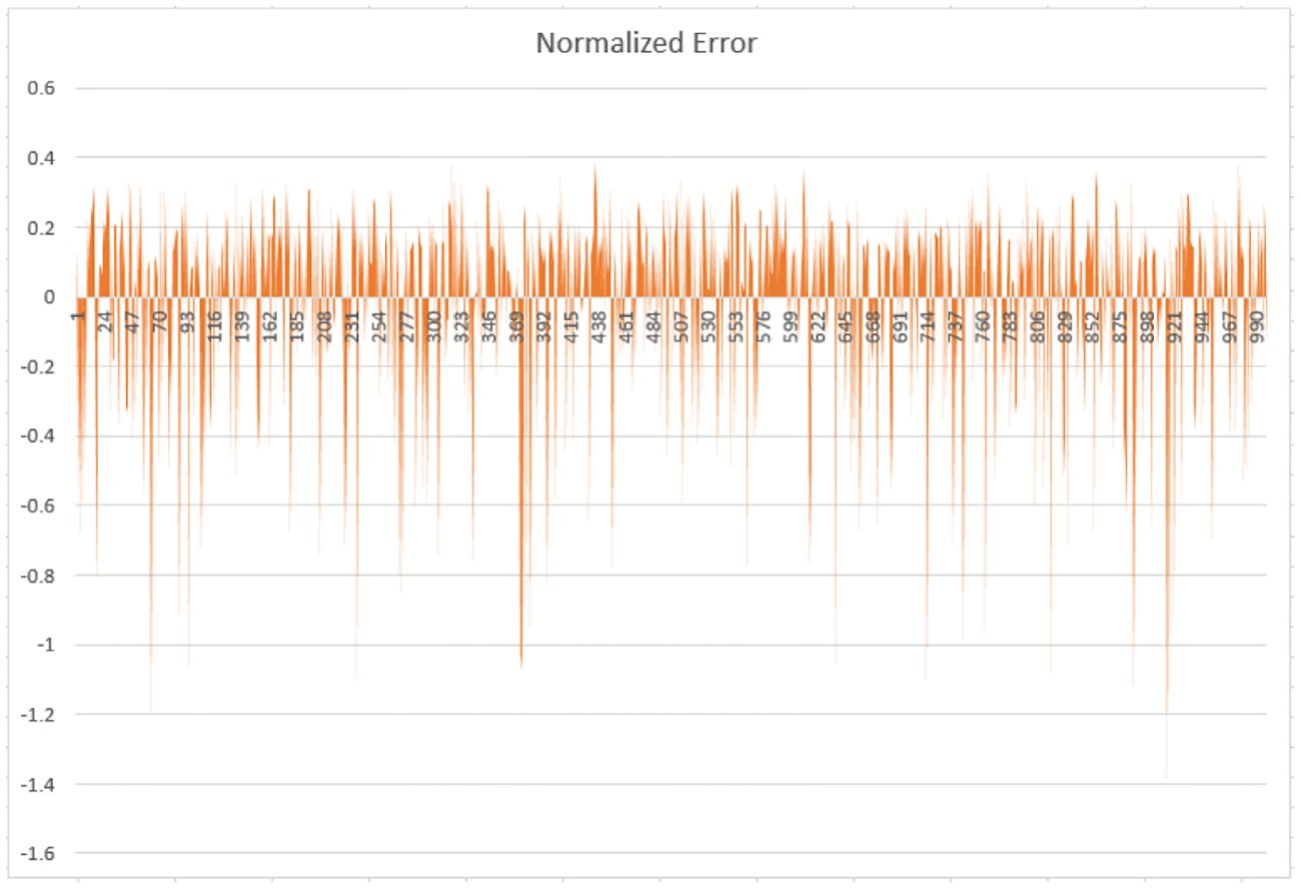
Normalized error based on absolute error in case of moving average 60.

The normalized error of the credit workload is plotted against the time units showing that in most cases, the error is below a threshold value, as shown in Fig 11. In some cases, the result goes beyond the lower limit of the threshold because of the absolute error instead of root mean square deviation. Our approach, when applied to a workload eradicate seasonality, irregularity, and trend component and shows the general trends as expected with a certain degree of inaccuracy.

**Fig 12:**
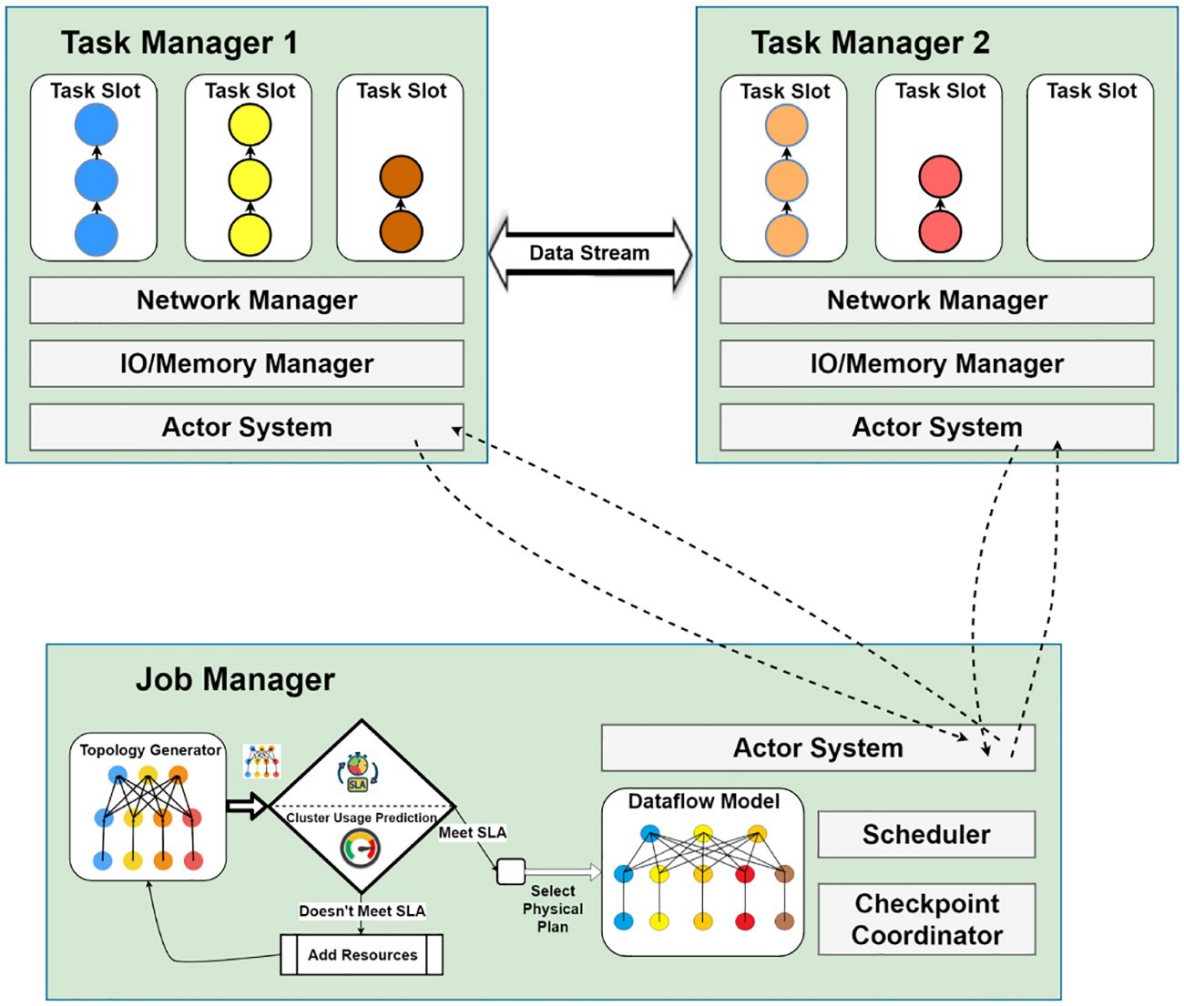
Use case scenario: Topology generator has given a default dataflow graph of map operator with parallelism 3 and reduce operator with parallelism 4. The system increases the parallelism of reduce task to 5 in order to achieve a higher QoS. Adapted from [10].

Although the prediction module can generate prediction all the time, to show its impact on QoS of user applications, another set of experiments are designed. The evaluation of the proposed TRS system was done through a use case scenario where the topology generator has given a default dataflow graph of map operator with parallelism 3 and reduce operator with parallelism four as shown in Fig 12. The scenario is described in details in our previous work [10]. The extended version of the module checks for the QoS targets of SLA agreement and available resources on a cluster. If the system does not achieve the QoS goals, it will request the resource manager for additional resources and repeat the process. Assuming the generated topology fulfills all QoS goals, which would gratify the SLA agreement, a physical execution plan is created by the data flow module, with a reformed parallelism of 3 for the map operator and 5 for the reduce operator. This increase in the reduce operator parallelism is due to the reason that reduces operator impedes in this use case. It is the responsibility of the job manager to transmit a signal and assign the task to available task slots on the registered task managers. In our use case scenario, the blue, yellow, and brown operators are assigned to task manager 1, while burlywood and orange operators are assigned to task manager 2.

The system’s hardware and software specifications are listed in Table 3. We use Apache Flink as a testbed for the evaluation of the system, as it is one of prominent distributed stream processing engines, which provides API for the ease of end-users and system administrators. We conducted our experiment using a virtual machine-based cluster and Ganglia was installed on the cluster. A total of three cases utilize 2 vCPUs, and 2 GiB memory respectively. Ganglia were selected to be used for evaluation purposes. Ganglia monitor the performance of the cluster as a whole as well as each machine’s performance and usage. A YARN cluster with default parallelism of 3 was used as the base cluster to execute applications over it.

**Table 3:** Cluster configuration.

Hardware / Software	Configuration
Cluster	Virtual Machine Cluster
Nodes	2 vCPU, 2GB
Number of Instances	3
Flink	Version 1.5
Ganglia	Version 3.7.2

**Fig 13:**
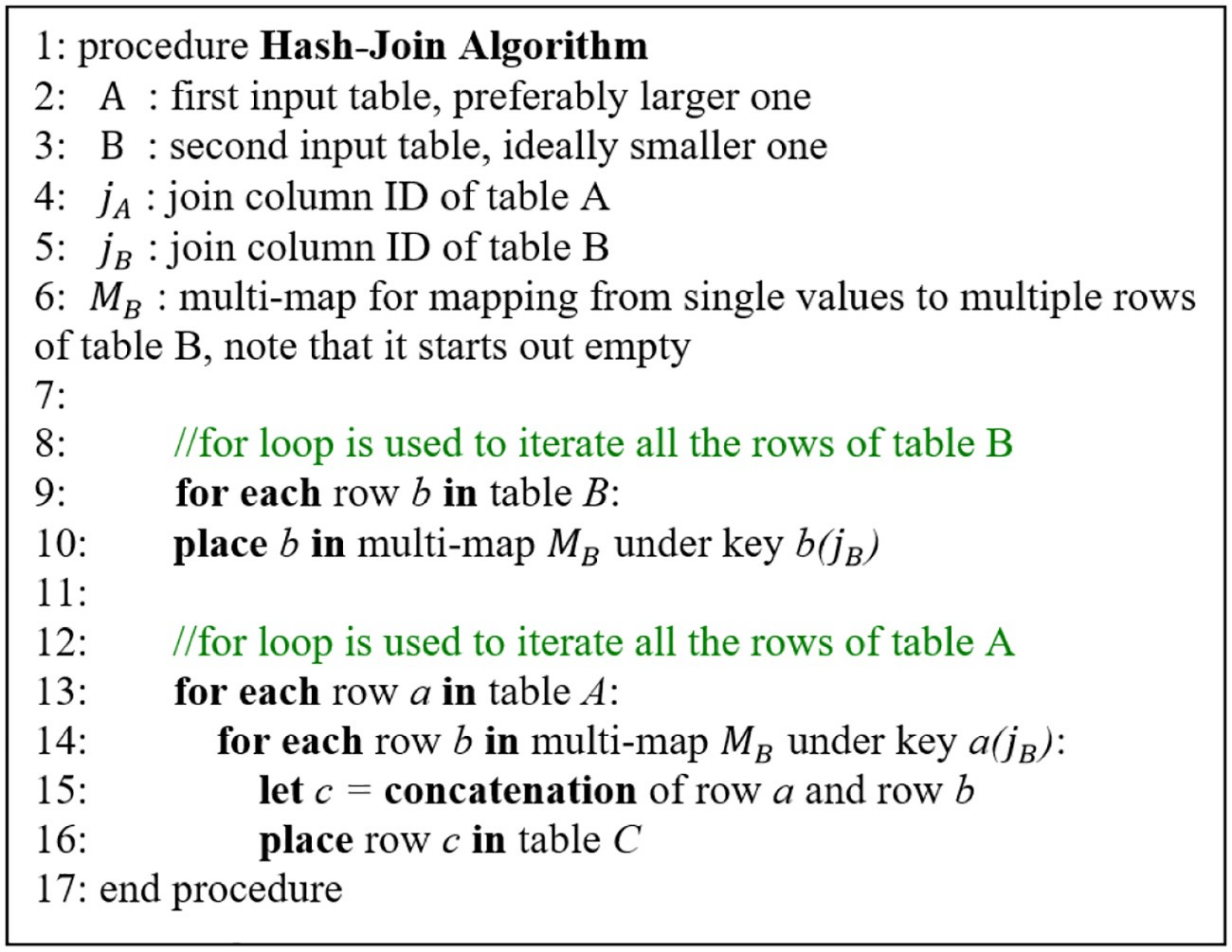
Pseudo-code of hash join algorithm: Join one small and one large table to create multi-map with the ability of hash-based lookup or search.

Furthermore, we ran an Apache Flink equivalent implementation of Hash join algorithm program in order to conceptualize that the proposed system has the ability to work with both the streaming and batching jobs accordingly. The pseudo-code of the algorithm is as shown in Fig 13. A Hash join alludes to a type of join command, wherein one table is designed to be compact to fit into the memory, while the other, larger table that cannot fit is read from a disk instead. The hash join algorithm consists of two operations, Hash phase and Join phase. Hash phase creates a multi-map from one of the two tables, preferably the smaller table, to minimize its memory size and creation time. Its creation process is mapping from each join column value of the table to all the rows that contain it. The multi-map must have the ability to support hash-based lookup in order to scale better as compared to linear search. Hash phase scan for the matching rows through looking in the multi-map and join the rows accordingly. In other words, this program is a distinctive kind of join command, which firstly obtains the location of the hash input table as well as the input data stream table, then invokes the join command to combine the hashing and the data stream as needed.

**Fig 14:**
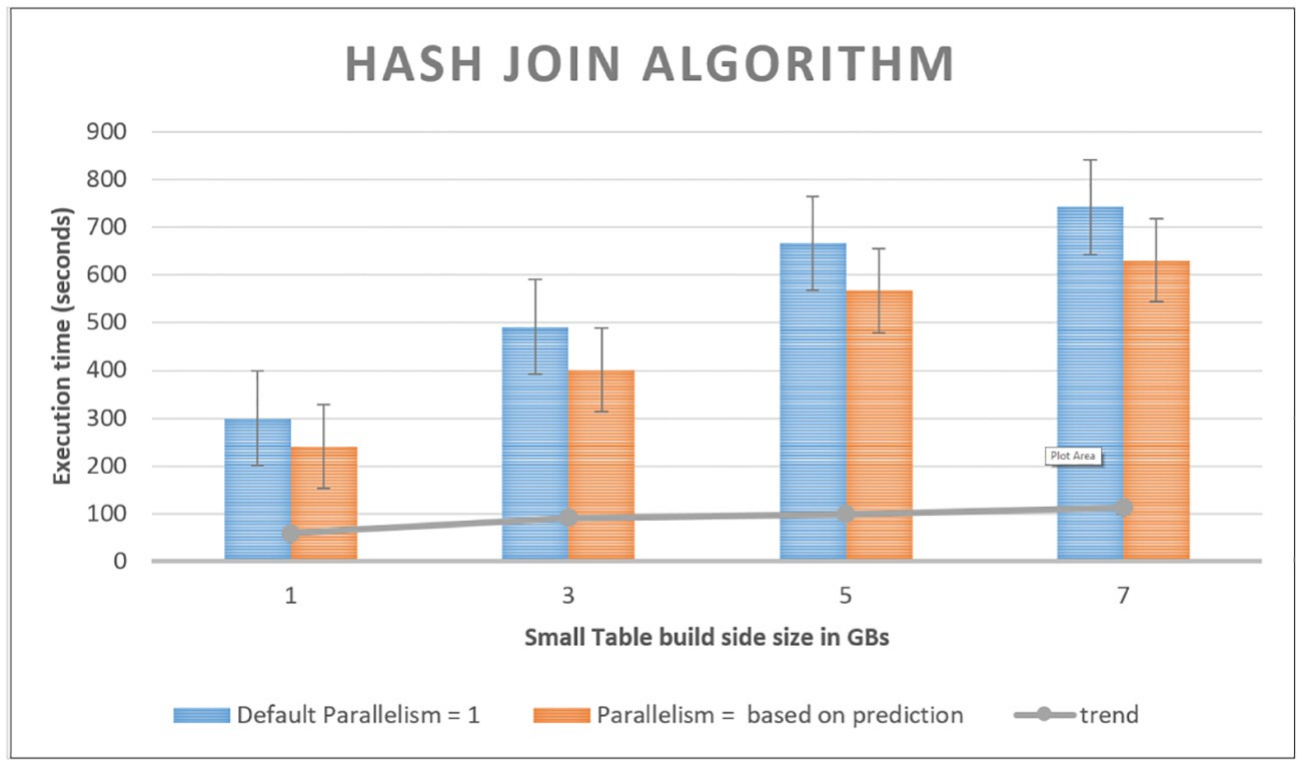
Execution time vs. build size: Execution time of hash join with one data set varying from 1GB to 7 GB and the other is kept constant at 10 GB.

We benchmark the performance of the system with parallelism 1 as default parallelism and compare it with prediction-based topological changes accordingly. The smaller table size is varied from 1 to 7 GB, while the larger table is kept constant at 10 GB. Fig 14 shows the average execution time of five runs of the hash join application where one (smaller in size) dataset is used as hash dataset to be joined with a larger dataset as detailed earlier. The join with one Gigabyte is pure in-memory join. The other joins spill data to disk partially. The results show that performance remains stable, until the hash table fits into memory, and gracefully decrease as the hash join function starts spilling data into disk. Our proposed TRS system has the ability to outperform the default system with the increase in the input data stream. The system shows significant improvements for the prediction-based topology configuration of the system. We plan to implement the system on top of the other distributed streaming processing systems, design experiments/use-cases and evaluate the system more thoroughly in the near future.

**Fig 15:**
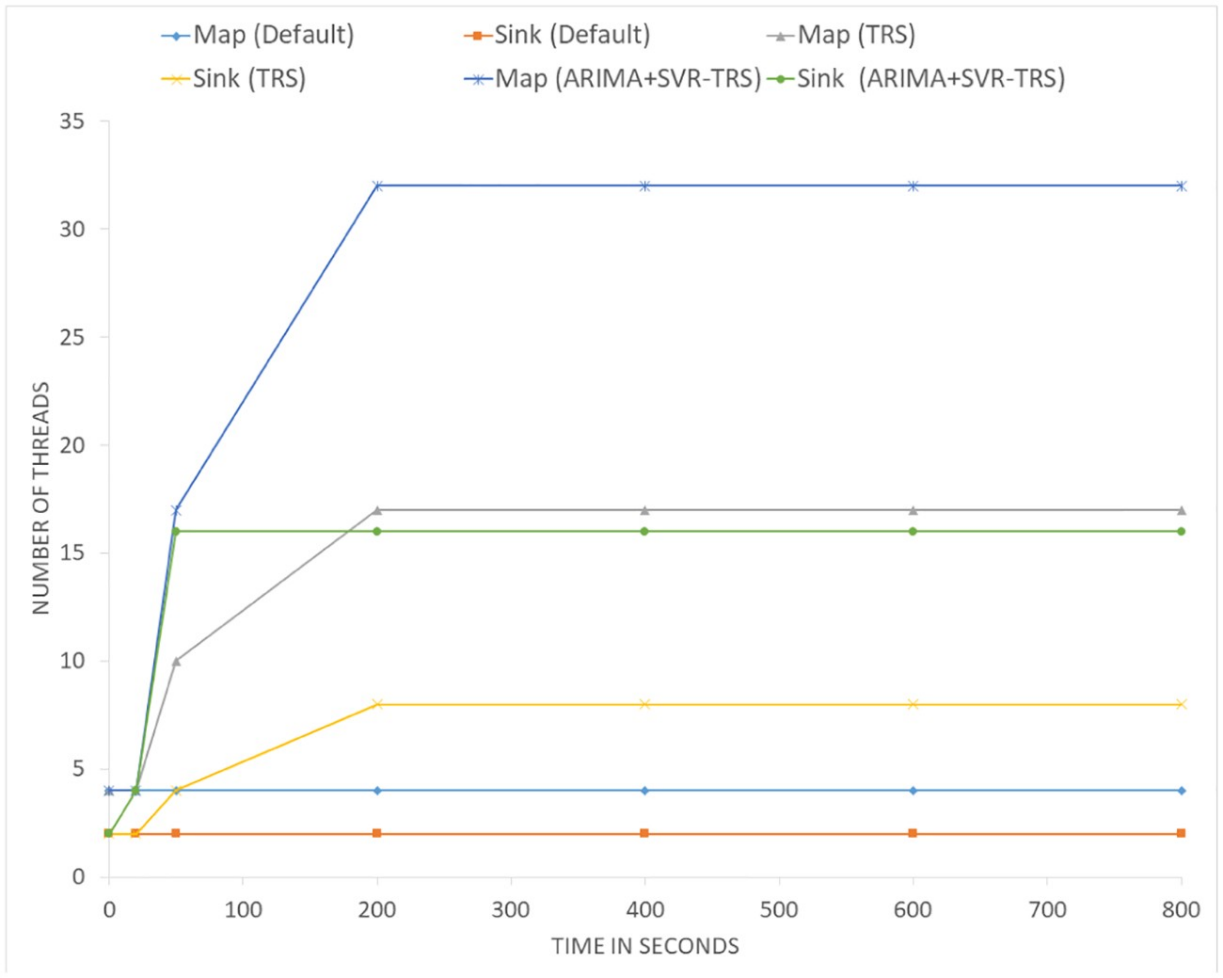
Comparing the default parallelism, TRS parallelism, and ARIMA+SVR TRS parallelism based on the incoming workload using Hash-Map Implementation with varying data set.

In order to demonstrate the generality of the scheme with varying number of parallel threads, we plotted the default parallelism of the Apache Flink, ARIMA based TRS, and the decisions taken by ARIMA+SVR TRS optimization. For this set of experiments, we averaged the values of over ten experimental runs and plotted the values in Fig 15. The default number of threads was set to four for the Map operator and two for the Join operator. The graph shows the number of threads running for the Map and Sink operator over time from the start until the solution reached a point of convergence. In the case of the default system, the number of Map operator’s threads stays at four and Sink operator’s thread stays at two throughout the entire lifespan of the experiment. After applying the TRS with an initial under-provisioned configuration, it changes the number of threads for Map operator to 10 and Sink operator to four to cope with the changes in the incoming workload. The system converges to the value of 17 for the Map operator and 8 for the Sink operator. In case of the hybrid model of SVR and ARIMA based TRS system, the map operator climbs up to 17 threads in a single step and then converges on 32 threads running in parallel. Finally, the Sink operator jumps to four and then converges to 16 threads running in parallel. Note that the small table has 17 sub-partition and the TRS system converges to it in just after two scaling decisions by correctly estimating the optimal parallelism in two steps.

## Related work

Cloud computing popularized the big data technologies to a new level with providing services online in a pay-as-you-go manner such as SPaaS (Streaming processing-as-a-service). With the number of enterprises offering cloud-based solutions to end-users and other enterprises, there has been a boom in the volume of data, creating interest of researchers from both industry and academia in big data analytics, streaming application, and social networking applications. This also has renewed the concern of QoS in streaming application, calling for adequate solutions that have the ability to adapt to workload changes. Basically, the problem can be tackled in two ways: workload prediction and system adaptation.

Workload prediction in cloud technologies is a well-researched topic. Many peers in the field have presented a variety of prediction models: one example includes, a pattern matching technique that is presented for grid-like workloads in cloud-based systems by finding similar occurrences in the past [26]. Event-aware workload prediction by Sladescu et al. [27] used ANN to predict workload burst in their proposal, Gong et al. [28] presented a mechanism to accurately predict the resources required in keeping with the application workload prediction. Islam et al. [29] applied ANN & linear regression based prediction system to develop resource management and provisioning strategy. Tran et al. [30] make use of the ARIMA model in order to predict the workload on servers. It targets long-term prediction up to 168 hours, whereas we target short term forecasting to be updated about workload changes at all time. Furthermore, we use the predictions to achieve QoS targets and maintain SLA constraints accordingly. A look-ahead resource allocation algorithm [31] applies ARMA model to predict workload in clouds to minimize cost. It predicts workload in accordance with the limited horizon, whereas our work focuses on achieving QoS targets to meet SLA constraints. Stochastic models are linear models with bounded capability to predict nonlinear data. In order to effectively predict nonlinear data, a number of researchers both in academia and industry used support vector machines (SVMs) such as artificial neural networks (ANNs) to predict horological and time series data in the past decade [32–34]. These models are machine-learning techniques that have been successfully applied in regression, classification, and forecasting. SVM can be divided into support vector classification (SVC) and support vecotor regression (SVR) that try to solve classification and regression problems, respectively. To improve prediction accuracy in time series forecasting, Choubin et al. [35] exemplify the effectiveness of adaptive neuro-fuzzy inference system (ANFIS) model in forecasting the SPI across different time scales. As opposed to ANFIS, ARIMA+SVR is a combination of linear and nonlinear model which is more suitable for time series workloads. Apart from that, ARIMA-ANN, coupling discrete wavelet transform (WA) and artificial neural networks (ANNs) as WA-ANN, multiple linear regression (MLR), multiple nonlinear regression (MNLR), HMM-based models, and combined HMM-Fuzzy models are also effective forecasting tools among the hybrid models [36–38].

The relevancy of steaming frameworks has been on the rise, resulting in an increase in projects focused on exploiting parallelism in stream processing. Apache S4 [39], Strom [3], and Flink [7] illustrate programs and queries as directed acyclic graphs (DAGs) with parallel operators. S4 allows the scheduling of parallel instances of operators, but cannot control said operators as a result. Storm permits its users to specify a parallelization level, while simultaneously supporting stream partitioning based on key intervals; however, it also ignores the operator’s states and has limited runtime scalability. System S [40] provides intra-query parallelism by way of a fine-grained subscription model able to express all sorts of stream connections but does not have an automated manager for the said mechanism. Hizrel [41] proposed a solution; A MatchRegex operator allows System S to discern tuple patterns in parallel. The approach does not factor in dynamic repartitioning and state as specific to an automata-based pattern detection mechanism. Stromy [42] utilizes consistent hashing and a logical ring to situate these new nodes, once the scale-out process has been completed. However, it omits congestion as a factor, as opposed to our proposed TRS system. Pattern-sensitive partitioning model [43] uses time series analysis to predict the incoming workload and estimates the parallelism of operations on the basis of queuing theory. This model is capable of attaining a high degree of parallelism for event patterns that could only be persistently detected in a sequential manner or at a lower parallelization degree. One study considered the giddiness of resource performance to maintain the throughput of the application at minimum resource cost using a heuristic resource adjustment method [44]. The proposed approach uses two greedy heuristics, centralized and sharded, which make use of the variable-sized bin packing algorithm. Multi-Objective Hybrid fruit fly Optimization (MOHFO) [45] adopted Bald Eagle Search (BES) optimization behaviour to amplify the searching ability for fruit fly optimization algorithm to achieve SLA-aware dynamic resource management in cloud data center. It follows a dynamic virtual machine consolidation and deployment scheme to attain a trade-off among resource wastage and SLA violations while TRS refine topology using workload prediction as heuristics. Profiling-based server consolidation framework [46] tries to minimize the number of physical machines used in data centers, while SLA into account using n integer programming model. It forecasts the micro architecture level interference through offline profiling phase. Zeitler and Risch [47] proposed a parasplit operator, intended as a partitioning stream statically based on a cost model, providing a customized stream splitting for the scalable execution of continuous queries over massive data streams. Alternatively, our proposition determines the parallelization level at runtime, according to established performance metrics. Backman et al. [48] segregated and spread operators across the various nodes within the stream processing framework to decrease the processing latency through load balancing in accordance with the simulated estimation of latency. They attained their latency reduction goals through their parallelism model, optimized by the latency-oriented operator scheduling procedure coupled with the diversification of the computing node responsibilities. StreamCloud [49] fashions elasticity into the Borealis Stream Processing Engine [50], and utilizes a query compiler to convert high-level queries into graphs of relational algebra operators, whilst utilizing a hash-based parallelization designed for the semantics of joins and aggregates. It alters the parallelism level by dividing queries into sub-queries while utilizing a balancing feature to regulate resource usages. Auto-parallelization [51] solves the profitability issue associated with the automatic parallelization of all-purpose distributed data stream processing applications. Their proposed solution can dynamically moderate the number of channels used to achieve high throughput and high resource utilization. In addition to its ability in handling partitioned stateful operators through run-time state migration. Whereas our work takes the workload into account and refines topology of the system to meet QoS targets of applications.

Heinze et al. [52] proposed an online parameter optimization method, enabling the system to provide monetary compensation to obtain the offered QoS. It focused on latency and policy rather than throughput and mechanism. Reactive-Scaling [53] offers a flexible elastic strategy for applying constraints over latencies in a scalable streaming framework while lowering resource footprints. Their queueing theoretic latency model provides a latency guarantee by adjusting the task-wise level of parallelism in a fixed size cluster. It should be noted though that our proposed methodology can be used as a black box within both systems [52] [53]. Mai et al. [54] presented a novel control-plane design with the ability to support constant monitoring and feedback in order to enable the systems to reconfigure dynamically. They clouts the key understandings of embedding control-plane messages in data channels to gain low latency and introduce asynchronous execution of policies to avoid global synchronization. Dhalion [55] addresses the issue of the task tuning of various configuration to achieve service level objectives and its maintenance in the presence of unpredictable changes in the underlying environment. The authors implemented their proposed system on top of twitter Heron and demonstrated its scaling capabilities accordingly. Finally, Fang et al. [56] addressed the problem of poor balancing in the presence of workload variance through key-based workload partitioning and tries to dynamically assign the workload to operators. They formulate the rebalancing operation as an optimization problem with objectives of diminishing state migration cost, controlling size of routing table, and balancing the work among worker nodes. While this work can handle short-term distribution fluctuation, our proposed algorithm has the ability to adapt to long-term, seasonal, and unplanned workload imbalances as well.

## Concluding remarks and future directions

There are growing concerns about the QoS of recent cloud services like stream-processing-as-a-service. With the increase of enterprises shifting from legacy systems to recent cloud technologies, competitiveness grows day by day. In such a competitive environment, the service providers need to focus on the QoS now more than ever to maintain their SLA agreement. One of the key factor affecting the QoS is variability in workload, as the systems need to be able to adapt accordingly. In order to thwart the problem, we proposed TRS system, a topology refining solution for stream processing systems, based on workload prediction mechanism. The prediction is made through a model based on a combination of SVR and ARIMA models with fine adjustments to make it work on the fly. The idea behind the proposed system is to increase the overall performance by keeping the topologies optimized all the time according to incoming workload, while still being able to satisfy QoS targets to maintain SLA constraints. In the next step, the authors plan to explore more efficient and promising ways to predict incoming workload such as different combinations of Particle Swarm Optimization (PSO), SVR, ARIMA, and Artificial Neural Network (ANN). Furthermore, we want to consider options to implement the TRS scheme on top of other state-of-the-art distributed streaming processing frameworks and design experiments and use-cases to evaluate the system more thoroughly.

## Supporting information

S1 File Data set1: The dataset used in the evaluation of the proposed system. This file contains a subset of the NYC TLC dataset used in evaluation of the proposed system.
(CSV)

S2 File Data set2: The dataset used in the evaluation of the proposed system. This file contains the Statlog German credit card dataset used in evaluation of the proposed system.
(RAR)

S3 File Code-Backup: The coding backup of the proposed system. This zip file contains the code-backup of the proposed system and its related files.
(ZIP)
